# The association between prescription change frequency, chronic disease score and hospital admissions: a case control study

**DOI:** 10.1186/2050-6511-14-39

**Published:** 2013-08-01

**Authors:** Carolien GM Sino, Rutger Stuffken, Eibert R Heerdink, Marieke J Schuurmans, Patrick C Souverein, Toine (A) CG Egberts

**Affiliations:** 1 University of Applied Sciences Utrecht, Research Centre for Innovation in Healthcare, Bolognalaan 101, 3584 Utrecht, CJ, The Netherlands; 2 Department of Pharmacoepidemiology and Clinical Pharmacology, Utrecht University, Faculty of Science, Utrecht, The Netherlands; 3 Department of Clinical Pharmacy, Tergooi Hospitals, Blaricum/Hilversum, The Netherlands; 4 Department of Rehabilitation, University Medical Centre Utrecht, Nursing Science and Sports, Utrecht, The Netherlands; 5 Department of Clinical Pharmacy, University Medical Centre Utrecht, Utrecht, The Netherlands

**Keywords:** Prescription changes, Prescription change frequency, Hospital admission, Chronic disease score

## Abstract

**Background:**

The aim of this study was to assess the association between prescription changes frequency (PCF) and hospital admissions and to compare the PCF to the Chronic Disease Score (CDS). The CDS measures comorbidity on the basis of the 1-year pharmacy dispensing data. In contrast, the PCF is based on prescription changes over a 3-month period.

**Methods:**

A retrospective matched case–control design was conducted. 10.000 patients were selected randomly from the Dutch PHARMO database, who had been hospitalized (index date) between July 1, 1998 and June 30, 2000. The primary study outcome was the number of prescription changes during several three-month time periods starting 18, 12, 9, 6, and 3 months before the index date. For each hospitalized patient, one nonhospitalized patient was matched for age, sex, and geographic area, and was assigned the same index date as the corresponding hospitalized patient. We classified four mutually exclusive types of prescription changes: change in dosage, switch, stop and start.

**Results:**

The study population comprised 8,681 hospitalized patients and an equal number of matched nonhospitalized patients. The odds ratio of hospital admission increased with an increase in PCF category. At 3 months before the index date from PCF=1 OR 1.4 [95% CI 1.3-1.5] to PCF= 2–3 OR 2.2 [95% CI 1.9-2.4] and to PCF ≥ 4 OR 4.1 [95% CI 3.1-5.1]. A higher CDS score was also associated with an increased odds ratio of hospitalization: OR 1.3 (95% CI 1.2-1.4) for CDS 3–4, and OR 3.0 (95% CI 2.7-3.3) for CDS 5 or higher.

**Conclusion:**

The prescription change frequency (PCF) is associated with hospital admission, like the CDS. Pharmacists and other healthcare workers should be alert when the frequency of prescription changes increases. Clinical rules could be helpful to make pharmacists and physicians aware of the risk of the number of prescription changes.

## Background

Medication-related problems are responsible for 3–10% of acute hospital admissions, of which approximately half are potentially preventable [1-11]. Hospital admissions can lead to additional functional decline [12,13], unintentional harm [14], and increased costs. Medication monitoring and management are methods used to avoid medication-related complications.

In 2008, the Dutch HARM study group established seven independent risk factors for medication-related hospital admissions: (a) impaired cognition, (b) four or more diseases in the patient’s medical history, (c) dependent living situation, (d) impaired renal function before hospitalization, (e) non-adherence to medication regimen, (f) the use of five or more medications at the time of admission (polypharmacy), and (g) age over 65 [11]. In the industrialized world, the proportion of the population that is 65 years or older is rapidly increasing. Elderly patients more often suffer from multiple morbidities, use more medications, and are treated by more healthcare professionals than younger patients [15]. Drug consumption is three times higher among people aged 65 years or older, and four times higher in people aged 75 years or older, than it is in people younger than 65 years. The majority of these drugs are taken chronically (http://www.SFK.nl). The increased use of prescription drugs by the elderly is a consequence of their longer lifespan, their increasing use of health services, and the availability of new drugs [16]. From a clinical perspective, prescription changes are a risk factor for medication-related hospital admission. During the course of a disease, it may be necessary to change the dosage of medication, to switch to a similar medication, to temporarily withdraw the drug, or to start a new drug. With the exception of the study of Koecheler [17], who reported ‘medication regimen changes in four or more times during the past 12 months’ to be one of the six prognostic indicators for identifying ambulatory patients who need pharmacist monitoring, there have been no other studies that evaluated the association between the number of prescription changes and hospital admission. For this reason, we investigated whether the frequency of prescription changes is associated with hospital admission, and, if so, whether the strength of this association changes in the months before hospital admission.

The Chronic Disease Score (CDS), a well-established instrument to predict hospital admission, measures comorbidity on the basis of the 1-year pharmacy dispensing data for 17 therapeutic groups of somatic medications intended for chronic use [18]. The latter makes the CDS a static instrument. In contrast, the Prescription Changes Frequency (PCF) is based on prescription changes over a 3-month period.

The objectives of this study were (1) to assess the association between the PCF and hospital admission at different times before admission and (2) to compare the PCF with the CDS for predicting hospital admission.

## Methods

### Study design and setting

This retrospective, matched case–control study used with permission data from the Dutch PHARMO Record Linkage System (RLS) (http://www.pharmo.nl). The PHARMO RLS includes the dispensing records of community pharmacies linked to hospital discharge records. It consists of a representative sample of more than 200 community pharmacies in more than 50 regions throughout the Netherlands and is representative for the Netherlands [19]. It currently includes data for more than 2 million residents (12% of the Dutch population) regardless of the type of medical insurance. The computerized pharmacy dispensing records contain information about drugs dispensed, dispensing date, prescribing physician, amount of drug dispensed, and prescribed dosage regimen. Patient information includes sex and date of birth. Each patient is assigned an anonymous unique patient identification code and each medication is also given a unique code, according to the Anatomical Therapeutic Chemical (ATC) classification system. This makes it possible to track drug therapy and changes in drug therapy over time. The database does not record the indication for which a medicine is prescribed and neither does it include all medications used because non-prescription products can be purchased over-the-counter.

### Cases and controls

Initially, 10,000 patients who had been hospitalized for the first time of possible repeated hospitalizations between July 1998 and June 2000 were randomly selected from the PHARMO RLS. The date of hospital admission was considered the index date. Each hospitalized patient was matched by age on birthday, sex, geographic area per pharmacy catchment area with a control patient who was assigned the same index date. Patients were included if medication data were available for at least 24 months before the index date.

### Prescription change frequency

A prescription is defined as one medication order. PCF is defined as the number of prescription changes made during a 3-month period, without distinguishing between intentional and unintentional changes. Four different types of prescription changes were distinguished: (1) change in dosage, (2a) product switch, (2b) generic brand switch, (2c) therapeutic switch, (3) stopping medication, and (4) starting medication (Table 1)*.* As we were interested in whether the PCF affects hospitalization over time, we calculated the PCF score for both patients and controls at 18, 12, 9, 6, and 3 months before the index date*.* The duration of use of each drug was estimated by dividing the number of dispensed units by the prescribed daily dose. Drugs that had a theoretical end date beyond 18, 12, 9, 6, or 3 months before the index date were considered as being in use on these dates. Only drugs intended for systemic use were taken into account. PCF scores were categorized into 0 prescription changes (PCF 0), 1 prescription change (PCF 1), 2 or 3 prescription changes (PCF 2 or 3), and 4 or more prescription changes (PCF≥ 4).

**Table 1:** Classification of prescription changes

**Classification**	**Definition**
1. Change in dosage	Change in dosage means that, for the same drug, the daily dosage is increased or decreased (e.g., amitriptyline 25 mg changes in amitryptyline 10 mg or vv).
2a. Product formulation switch	Metoprolol 50 mg plain tablet instead of metoprolol slow release tablet (Selokeen ZOC®).
2b. Generic brand switch	Change to another product containing the same active substance with the same strength and the same dosage (e.g., atenolol 50 mg tablet (generic product) instead of Tenormin® 50 mg tablet (brand) or Renitec® 10 mg tablet (brand) instead of enalapril 10 mg tablet).
2c. Therapeutic switch	Change to another active substance within the same therapeutic group; the first four characters of the ATC classification are the same (e.g. amitriptyline (N06AA09) instead of citalopram (N06AB04) or fluoxetine (N06AB03) instead of citalopram (N06AB04)).
3. Stop	No continuation 90 days after one of the five control time points and no generic-brand substitution (1), product formulation switch (2) ortherapeutic switch (3).
4. Start	Start of a drug means prescription of a drug which had not been prescribed during the previous six months and which is not a generic brand substitution (1), product formulation switch (2) or therapeutic switch (3).

### Chronic disease score

The CDS is calculated on the basis of the use over 1 year of medications for 17 therapeutic groups of somatic medications. The CDS has been shown to be a valid measure of complications related to an individual patient’s burden of chronic somatic diseases and is clearly associated with the probability of being hospitalized [20-22]. To compare the PCF with the CDS, we calculated and categorized the CDS for the year preceding the index date into four categories: CDS score = 0, CDS score = 1 or 2, CDS score = 3 or 4, and CDS score 5 or higher.

### Statistical analysis

The strength of the association between the PCF score and hospital admission was calculated by comparing the number of patients and controls in each PCF category at 18, 12, 9, 6 and 3 months before the index date with forced entry univariate logistic regression analysis; outcomes are expressed as the odds ratio (95% CI), using PCF 0 as reference. To assess the effects of other patient or hospitalization characteristics, we performed stratified analyses with age (< 65 years ≥ 65 years), admission type (emergency or planned), CDS score, and polypharmacy (the use of five or more drugs concomitantly) as variables. To assess the strength of the association between the CDS score and hospital admission, the number of patients and controls per CDS category were compared (expressed as OR 95% CI), taking CDS 0 as reference. The nature of prescription changes was determined for each time period. The correlation between the PCF and the CDS was measured with a two-tailed Spearman‘s correlation coefficient. Statistical analyses were performed using SPSS 16.0 (SPSS, Chicago, IL).

## Results

The source population was a random sample of 10,000 patients admitted to a hospital and an equal number of matched non-admitted individuals (controls). Because 1319 matched patients had less than 24 months of exposure history available in PHARMO RLS, the final study population comprised 8681 patients and 8681 controls. The characteristics of the study population are displayed in Table 2. The mean age was 52.6 years (SD 21.8) and 58.7% of the participants were women. At the index date, 60.6% of the patients and 47.8% of the controls were using systemic medication; the mean number of drugs used at the index date was 3.0 for patients and 2.1 for controls. In both groups, the number of drugs used increased with age. The CDS was higher in the patients than in the controls. The most frequent reasons for prescription changes at all time points before the index date were stopping medication and changes in dosage (Table 3).

**Table 2:** Characteristics of hospitalized and non-hospitalized patients at the index date

**Characteristics**		**Hospitalized**	**%**	**Non-Hospitalized**	**%**
		**N=8681**		**N=8681**	
**Sex**					
	Male	3588	41.3	3588	41.3
	Female	5093	58.7	5093	58.7
**Age** (years at index date)					
	0 - ≥ 18	574	6.6	574	6.6
	>18 - ≥ 45	2737	31.5	2737	31.5
	> 45 - ≥ 65	2246	25.9	2246	25.9
	> 65 - ≥ 79	2218	25.6	2218	25.6
	> 79	906	10.4	906	10.4
**Number of medications**					
	0	3416	39.4	4534	52.2
	**1**	1794	20.7	2121	24.4
	2	985	11.3	872	10.0
	3	767	8.8	535	6.2
	4	544	6.3	302	3.5
	≥5	1175	13.5	317	3.7
**CDS category**					
	CDS score 0	3671	42.3	5206	60.0
	CDS score 1-2	1331	15.3	1287	14.3
	CDS score 3-4	1731	19.9	1415	16.3
	CDS score ≥5	1948	22.4	773	8.9
**Duration of hospitalization**					
	1 day	417	4.8		
	2-5 days	4374	50.4		
	> 5days	3890	44.8		
**Admission type**					
	Emergency	3966	45.7		
	Planned	4715	54.3		
**Admission for surgery**					
	Yes	4360	50.2		
	No	4321	49.8		

**Table 3:** The association between prescription change TYPE and hospital admission at different time points before index date

	**-18**					**-12**					**-9**					**-6**					**-3**				
	**H**	**NH**	***OR***	**CI 95%**		**H**	**NH**	***OR***	**CI 95%**		**H**	**NH**	***OR***	**CI 95%**		**H**	**NH**	***OR***	**CI 95%**		**H**	**NH**	***OR***	**CI 95%**	
**PC TYPE**																									
***Change in Dosage***	946	521	***1.4***	1.3	1.6	1183	699	***1.3***	1.2	1.4	1093	597	***1.4***	1.3	1.6	1162	639	***1.4***	1.3	1.5	1405	656	***1.5***	1.4	1.6
***Product Switch***	211	127	***1.6***	1.3	2.0	329	187	***1.6***	1.3	1.9	325	202	***1.5***	1.3	1.8	349	217	***1.5***	1.3	1.8	422	245	***1.6***	1.4	1.8
***Generic Brand Switch***	114	67	***1.7***	1.2	2.2	180	93	***1.8***	1.4	2.3	192	103	***1.8***	1.4	2.3	219	101	***2.1***	1.6	2.6	274	91	***2.8***	2.2	3.5
***Therap. Switch***	221	100	***1.9***	1.5	2.4	230	117	***1.7***	1.4	2.1	256	96	***2.3***	1.8	2.9	300	111	***2.3***	1.8	2.8	345	117	***2.6***	2.1	3.1
***Stop***	2735	1923	***1.3***	1.2	1.3	2961	1910	***1.3***	1.3	1.4	3122	1950	***1.4***	1.3	1.4	3122	1943	***1.4***	1.3	1.4	3102	2005	***1.3***	1.3	1.4
***Start***	162	61	***2.3***	1.7	3.0	136	76	***1.6***	1.3	2.1	157	61	***2.3***	1.8	3.1	186	75	***2.2***	1.7	2.8	227	71	***2.9***	2.2	3.7

H=hospitalized patients (N=8681), NH=Non=Hospitalized Patients (N=8681), OR=Odds Ratio, CI 95%= Confidence Interval 95%.

PC Type=Prescription Change Type.

The risk of hospital admission increased with the number of prescription changes. At 3 months before the index date, the likelihood of hospitalization increased with increasing PCF category: the odds ratio (OR) between patients and controls was 1.4 (95% CI 1.3-1.5) in the lowest PCF category (PCF 1) and 4.1 (95% CI 3.1-5.1) in the highest PCF category (PCF 4)*.* This was also true for comparisons for 18, 12, 9, and 6 months before index date (Tables 4 and 5).

**Table 4:** The association between prescription change frequency and hospital admission at different time points before index date

	**-18**					**-12**					**-9**					**-6**					**-3**				
	**H**	**NH**	***OR***	**CI 95%**		**H**	**NH**	***OR***	**CI 95%**		**H**	**NH**	***OR***	**CI 95%**		**H**	**NH**	***OR***	**CI 95%**		**H**	**NH**	***OR***	**CI 95%**	
**PCF Cat**																									
**0**	**6086**	6736	***1***	ref		5844	6524	***1***	ref		5788	6556	***1***	ref		5723	6570	***1***	ref		5591	6537	***1***	ref	
**1**	1631	1418	***1.3***	1.2	1.4	1731	1564	***1.2***	1.2	1.3	1720	1561	***1.3***	1.2	1.4	1751	1483	***1.4***	1.3	1.5	5591	1493	***1.4***	1.3	1.5
**2 or 3**	760	451	***1.9***	1.7	2.1	853	514	***1.9***	1.7	2.1	899	490	***2.1***	1.9	2.3	923	542	***2.0***	1.8	2.2	1031	560	***2.2***	1.9	2.4
**≥ 4**	204	76	***3.0***	2.3	2.4	253	79	***3.6***	2.8	4.6	274	74	***4.2***	3.3	5.4	284	86	***3.8***	3.0	4.8	316	91	***4.1***	3.1	5.1

H=hospitalized patients (N=8681), NH=Non=Hospitalized Patients (N=8681).

OR=Odds Ratio, CI 95%= Confidence Interval 95%.

PCF Cat=Prescription Change Frequency Category.

**Table 5:** The association between the chronic disease score and hospital admission

	**Indexdate**				
	**Hospitalized patients N=8681**	**Non Hospitalized patients N=8681)**	***OR***	**CI 95%**	
**CDS score**					
**0**	3671	5206	***1***	**ref**	
**1 or 2**	1331	1287	***1.04***	0.96	1.13
**3 or 4**	1731	1415	***1.27***	1.18	1.38
**≥ 4**	1948	773	***2.95***	2.71	3.23
*OR=Odds Ratio,*					
*CI 95%= Confidence Interval 95%,*					
*CDS =Chronic Disease Score at indexdate*					
*ref= reference*					

The risk of hospital admission also increased per CDS category. A higher CDS score was associated with an increased risk of hospitalization: OR 1.5 (95% CI 1.4-1.6]) for CDS 1–2, OR 1.7 (95% CI 1.6-1.9) for CDS 3–4, and OR 3.6 (95% CI 3.3-3.9) for CDS 5 or higher.

**Figure 1:**
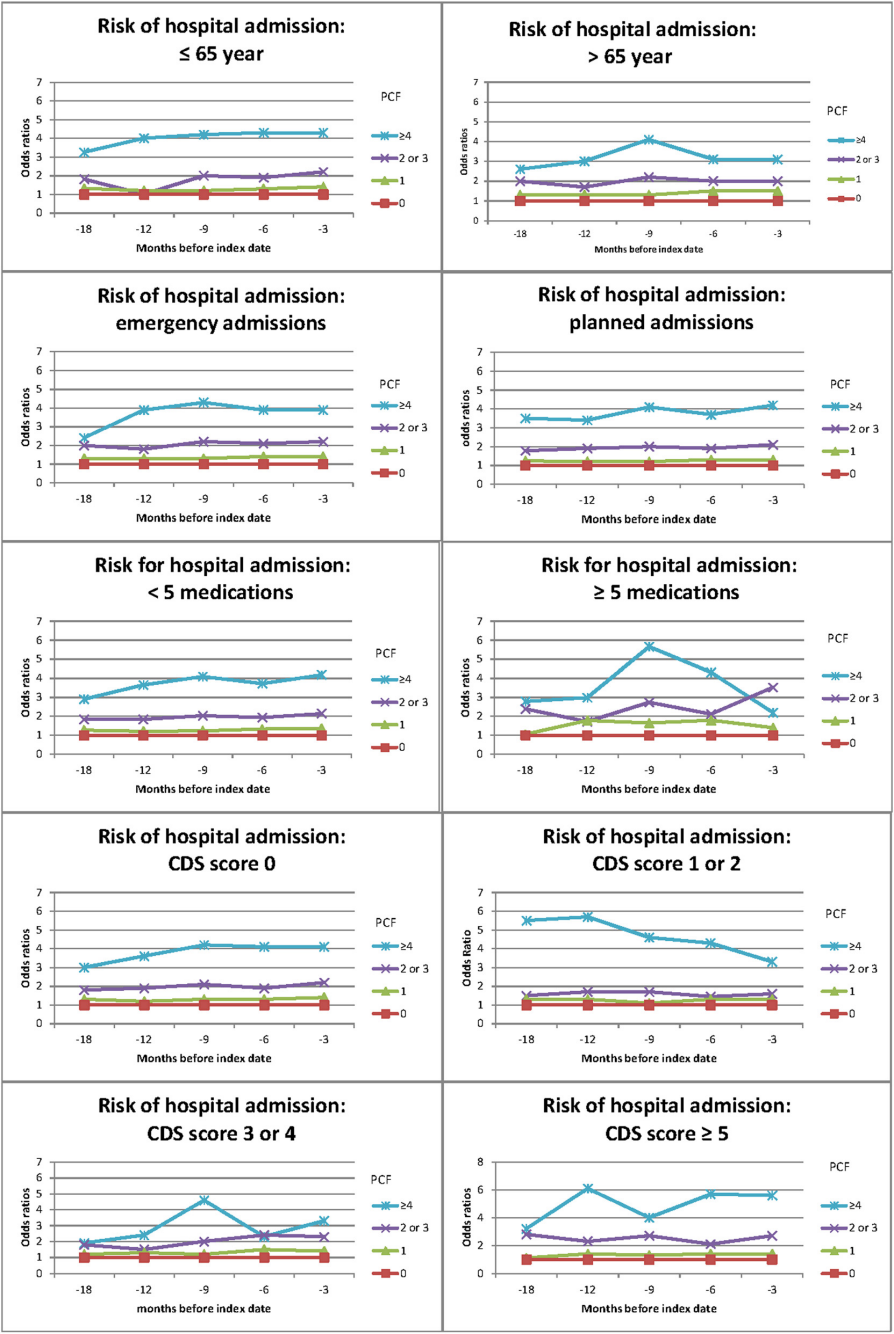
Stratification on age category, admission type, polypharmacy and CDS score.

Stratification by age (< 65 years ≥ 65 years), admission type (planned or emergency admission), CDS score, and polypharmacy resulted in comparable increases in OR with increasing PCF score. For participants on polypharmacy, the OR of PCF 4 or more decreased between 9 and 3 months before the index date, from 3.5 (95% CI 1.9-6.67) to 2.2 (95% CI 1.0-5.4)*.* When stratified by CDS, the likelihood of being hospitalized also increased with increasing PCF score (Figure 1)*.*

A two-tailed Spearman’ correlation coefficient showed a significant but poor correlation between CDS 0 and PCI 0 (0.019, *p*= 0.01) and CDS 5 or higher and PCI 4 or higher (0.027, *p*=0.01) and no significant correlation between CDS 1 or 2 and PCF 1 and CDS 3 or 4 and PCF 2 or 3 at 3 months before the index date.

## Discussion

The main finding of this study is that the frequency of prescription changes (PCF) is associated with an increased risk of hospital admission. We also confirmed the known association between the Chronic Disease Score (CDS) and hospital admission. While the PCF and CDS were both associated with hospital admission, the correlation between the two instruments was poor. The CDS measures comorbidity on the basis of the 1-year pharmacy dispensing data. In contrast, the PCF is based on prescription changes over a 3-month period. The results showed that the PCF within a three month period is comparable with the one year period of the CDS. Therefore, the PCF is more useful in practice.

We found that among patients with a low CDS score, an increasing number of prescription changes was associated with an increased risk of hospital admission. Stratified analysis of the CDS scores into the four categories confirmed this finding: at each CDS category, we found a comparable increase in the risk of hospitalization caused by the number of prescription changes.

Stratification by age (<65 or ≥65 year) and medication use (< 5 or ≥5 medications used) showed an increasing risk of hospitalization with increasing PCF (Figure 1). Several studies have reported age and polypharmacy as risk factors for hospital admission. We found that, based on PCF scores, even patients younger than 65 years and patients without polypharmacy were at increased risk of hospital admission. It is plausible that the risk was lower for planned than for emergency admissions, but this was not confirmed after stratification by type of hospitalization. Unexpectedly, patients on polypharmacy had a decreased risk of hospital admission: PCF 4 or higher decreased between 9 and 3 months before the index date. On the basis of this finding, the most common reason for prescription changes, namely, stopping medication, would appear to be protective against hospital admission in patients on polypharmacy. As we do not know which medications were stopped, this finding does not mean that stopping specific medications is protective.

The CDS has the disadvantage that it is based on information about medication history collected for at least 1 year prior to the event under investigation. We showed that it is possible to predict the risk of hospitalization on the basis of the number of prescription changes in 3 months. On the other hand, the CDS is based on the use for 17 therapeutic groups of somatic medications, whereas the PCF is based on all medications and thus requires detailed medication histories. The CDS was developed to measure a patient’s overall health status, but the PCF is not suitable for this. A potential weakness of the CDS, which was developed in 1992, is that it has never been adjusted to accommodate new medication classes, unlike the PCF, which is based on all medications used. Despite this, the CDS is still associated with hospital admissions.

### Limitations

This study has a number of limitations. The database does not provide information about the indication for which a drug is prescribed, so we cannot comment about the frequency of medication changes for specific indications. One could argue that more ill patients will have more prescription changes. However, this was not the aim of the study. The use of non-prescription medicines is not known as patients could also buy medications OTC. In addition, prescribers might not write out a new prescription each time drug use is changed. Because the PCF is based on dispensing data from community pharmacies, this would mean that the association between PCF and hospital admission might have been underestimated. As the data set used in this study covered the period between July 1998 and June 2000, it is possible, but unlikely, that since then the prescribing behavior of doctors has changed, influenced by medication reconciliation programmes, or indications for hospital admission might have become stricter, both of which would have led to overestimation of the association between PCF and hospitalization. While the Dutch PHARMO database is complete, it does not provide information about the socioeconomic status or compliance of patients or their health status (the controls might have been ill less often than the patients); however, as the controls were sampled independently of exposure status, these factors would not influence our results. Lastly, it was outside the scope of this study to distinguish between the different reasons for changing medication in greater detail. To our knowledge, besides the study of Koecheler *et al*. [17], no other studies have investigated prescription changes and the risk of hospital admission. Several other studies, like the HARM study, have described risk factors for medication-related hospital admission, but did not focus on prescription changes.

Further research should consider more detailed variables of the prescription changes like types of medications involved. In addition, it should be interesting to test the PCF model in a follow up study.

## Conclusion

This longitudinal study of a large group of patients over 24 months demonstrated that the frequency of prescription changes (PCF) over a 3-month period is associated with hospital admission, which suggests that the PCF could be used as an alternative to the CDS for predicting hospital admission. In the ambulant setting, the PCF score could function as a warning signal for an increased risk of hospitalization and as such contribute to medication safety programmes. The PCF might be particularly useful for older patients, who tend to use more medications. District nurses and social workers care should be alerted if the frequency of prescription changes increases in their patients. Community pharmacists can use the PCF as a clinical rule to facilitate early identification of potential drug-related problems. Further research is needed to determine the predictive value of the PCF in practice as a clinical rule.

## Competing interests

Possible conflict of interest: nothing to disclose.The department of Pharmacoepidemiology & Pharmacology has received unrestricted funding for pharmacoepidemiological research from GlaxoSmithKline, Novo Nordisk, the private-public funded Top Institute Pharma (http://www.tipharma.nl, includes co-funding from universities, government and industry), the Dutch Medicines Evaluation Board and the Dutch Ministry of Health.

## Authors’ contributions

All authors contribute the study conception and design and the study’s analytic strategy (CS-RS-EH-MS-PS-TE). PS prepared the database for analysis. CS has done the statistical data analysis, supported by EH and PS. CS and RS conduct the literature review and have written the drafting of the manuscript. Author MS and TE supervised the study and helped with critical revisions of the manuscript for important intellectual content. All authors read and approved the final manuscript.

## Pre-publication history

The pre-publication history for this paper can be accessed here:

http://www.biomedcentral.com/2050-6511/14/39/prepub
